# Global repositioning of transcription start sites in a plant-fermenting bacterium

**DOI:** 10.1038/ncomms13783

**Published:** 2016-12-16

**Authors:** Magali Boutard, Laurence Ettwiller, Tristan Cerisy, Adriana Alberti, Karine Labadie, Marcel Salanoubat, Ira Schildkraut, Andrew C. Tolonen

**Affiliations:** 1 1CEA, DRF, IG, Genoscope, Évry 91000, France; 2 CNRS-UMR8030, Évry 91000, France; 3 New England Biolabs, Inc., Ipswich, Massachusetts 01938, USA; 4 Université Paris-Saclay, Évry 91000, France; 5 Université d'Évry, Évry 91000, France

## Abstract

Bacteria respond to their environment by regulating mRNA synthesis, often by altering the genomic sites at which RNA polymerase initiates transcription. Here, we investigate genome-wide changes in transcription start site (TSS) usage by *Clostridium phytofermentans*, a model bacterium for fermentation of lignocellulosic biomass. We quantify expression of nearly 10,000 TSS at single base resolution by Capp-Switch sequencing, which combines capture of synthetically capped 5′ mRNA fragments with template-switching reverse transcription. We find the locations and expression levels of TSS for hundreds of genes change during metabolism of different plant substrates. We show that TSS reveals riboswitches, non-coding RNA and novel transcription units. We identify sequence motifs associated with carbon source-specific TSS and use them for regulon discovery, implicating a LacI/GalR protein in control of pectin metabolism. We discuss how the high resolution and specificity of Capp-Switch enables study of condition-specific changes in transcription initiation in bacteria.

## Abstract (web summary)

Bacteria may respond to a change in environment by using alternative transcriptional start sites. Here, the authors use a novel genome-wide capture and reverse transcription method to find substrate-specific start sites for hundreds of genes at single base resolution in *Clostridium phytofermentans*.

Bacteria translate environmental signals into cellular responses using a network of regulatory RNA and proteins that control genome-wide transcription patterns. Many of these regulators affect where RNA polymerase initiates messenger RNA (mRNA) synthesis at transcription start sites (TSS). As such, locating and quantifying changes in TSS usage is an important step to understand bacterial gene regulation. Here, we investigate TSS architecture in *Clostridium phytofermentans* ISDg, a soil bacterium that ferments plant biomass into ethanol, H_2_ and acetate^1^, and belongs to the *Lachnospiraceae* family that includes gut commensals with important roles in host nutrition^2^,^3^. This anaerobic mesophile metabolizes diverse plant components including cellulose, hemicellulose and pectin by tailoring expression of many carbohydrate-active enzymes (CAZymes) and other metabolic enzymes to the available substrate^4^,^5^. *C. phytofermentans* has a 4.8 Mb genome with 3,926 predicted protein-encoding genes^3^, and its ability to alter gene expression in response to carbon sources and other environmental cues is mediated by over 300 transcription regulator proteins^6^ and numerous non-coding RNA including metabolite-sensing riboswitches^7^.

We investigate genome-wide patterns of *C. phytofermentans* transcription initiation on heterogeneous plant substrates by demonstrating an approach called Capp-Switch sequencing. The initiating nucleotide of nascent mRNA is distinguished by a 5′ triphosphate (5′-PPP), which has been exploited for genome-wide TSS identification with dRNA-seq^8^ by depleting rRNA and other monophosphorylated transcripts using terminal exonuclease (TEX). dRNA-seq has been applied to diverse bacteria^9^,^10^,^11^,^12^,^13^, but incomplete and non-specific degradation of processed RNA requires TSS identification to be based on statistical comparison of read coverage in +TEX and −TEX samples. Capp-Switch avoids these problems by capturing and purifying 5′ mRNA fragments, which are reverse transcribed with template-switching to tagged cDNA for high-throughput sequencing (Fig. 1). The 5′-PPP of mRNA are modified by vaccinia capping enzyme (VCE) to bear a biotinylated guanosine cap that facilitates their capture and purification using streptavidin magnetic beads. Recently, TSS were identified by Cappable-Seq^14^ using VCE to add a desthiobiotin cap for bead-based capture of 5′ mRNA, which were then eluted from the beads and de-capped to ligate adapters for reverse transcription to tagged cDNA. Capp-Switch streamlines this approach by reverse transcribing the 5′ mRNA fragments using template-switching by Moloney murine leukemia virus reverse (MMLV) transcriptase^15^. Template-switching avoids adapter ligation and enables synthesis of 5′-tagged cDNA without releasing RNA from the beads, permitting use of an irreversible, biotinylated cap to increase RNA capture affinity. In all, we show Capp-Switch is a robust method that yields a genome-wide, strand-specific, quantitative map of TSS at single nucleotide resolution.

We apply Capp-Switch sequencing to define a genome-wide map of 9,457 TSS during *C. phytofermentans* growth on raw biomass, heterogeneous polysaccharides (cellulose, hemicellulose and pectin) and their constituent sugars. We use this TSS map to investigate features controlling gene regulation, such as RNA polymerase binding sites, 5′ untranslated region (UTR) structure, alternative promoters, operons and non-standard (leaderless and antisense) transcription. We identify sequence motifs associated with groups of TSS that are differentially expressed on specific carbon sources and show these motifs can be used to reconstruct transcription factor regulons. By integrating Capp-Switch data with an updated genome annotation, RNA-seq and proteomics, we discover novel transcriptional units (TU) and protein-encoding genes. Finally, we discuss how Capp-Switch sequencing can be applied as a general approach to explore transcription regulation in prokaryotes.

## Results

### General transcriptome features

**Figure 2:**
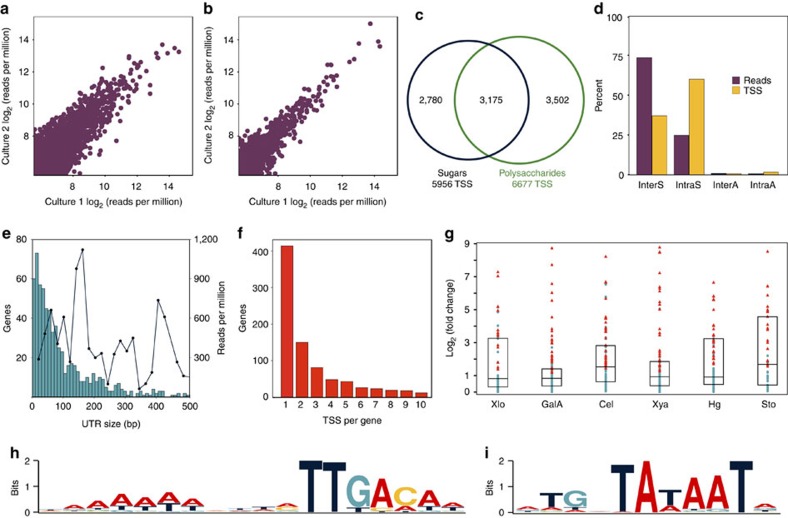
General features of TSS identification by Capp-Switch sequencing. Capp-Switch reproducibly quantifies TSS usage in duplicate (**a**) glucose (4,399 TSS; *R*^2^=0.96) and (**b**) stover (1,532 TSS; *R*^2^=0.99) cultures. (**c**) Venn diagram showing overlap of TSS identified in at least one monosaccharide and one polysaccharide or biomass treatment. (**d**) Percentage of reads (purple) and TSS (yellow) classified as InterS, IntraS, InterA or IntraA summed across treatments. (**e**) The length of most 5′ UTR (primary TSS to start codon) is <100 bp (blue bars with left *Y* axis), but UTR length does not correlate with expression strength (black line with right *Y* axis). TSS strength is the average reads per million for all TSS in a 20 bp 5′ UTR size interval. Results show glucose data. (**f**) Distribution of the number of InterS TSS per gene for data summed across treatments. (**g**) Genes with substrate-specific TSS are often differentially expressed. The *Y* axis is the absolute value of log_2_ (RPKM substrate/RPKM glucose) from RNA-seq for all genes with InterS TSS specific to that substrate. Substrates are xylose (Xlo *n*=50 genes), galacturonic acid (GalA *n*=146 genes), cellulose (Cel *n*=94 genes), xylan (Xya *n*=91 genes), pectin (Hg *n*=119 genes) and stover (Sto *n*=48 genes). Symbols: red triangles are differentially expressed genes, blue circles unchanged genes, box shows median and interquartile range. Promoter regions upstream of TSS expressed on three sugars and polysaccharides show consensus (**h**) −35 and (**i**) −10 motifs recognized by RNA polymerase.

Capp-Switch sequencing quantified TSS with high reproducibility between duplicate model substrate (Fig. 2a) and raw biomass (Fig. 2b) cultures. We identified 9,457 TSS across treatments (Supplementary Data 1), one-third of which were expressed in both sugar and polysaccharide cultures (Fig. 2c). Most reads (74%) contribute to InterS TSS (Fig. 2d), which we observed upstream of 898 genes. Among these, 687 genes (77%) are predicted to start operons^16^ (Supplementary Data 2), supporting these operon predictions and the existence of many sub-operons. The 5′ UTR, spanning from the primary TSS to the start codon, is less than 100 bp for most genes, but there is no correlation between 5′ UTR length and TSS strength (Fig. 2e). Studies in other bacteria report many leaderless mRNA without 5′ UTR and ribosome binding sites (RBS)^11^. Four per cent of InterS TSS are potentially leaderless in *C. phytofermentans*, but these genes generally have another upstream TSS and retain a typical RBS similar to highly expressed *C. phytofermentans* genes (Supplementary Fig. 1).

Most genes were expressed from a single, primary TSS on all substrates (Fig. 2f), but 191 (21%) genes altered their primary TSS in response to carbon source. Further, genes with substrate-specific InterS TSS are often differentially expressed on that carbon source (*χ*^2^ test, *P*<0.01 for all substrates relative to glucose) (Fig. 2g), supporting that changing TSS is a widespread means of transcription regulation. In total, more than a thousand TSS are specific to each polysaccharide (Supplementary Fig. 2A). Xylan-specific (Supplementary Fig. 2B) and pectin-specific (Supplementary Fig. 2C) TSS are primarily associated with carbohydrate metabolism genes, while the most abundant functional category of cellulose-specific TSS is prophage genes (Supplementary Fig. 2D). The *C. phytofermentans* genome includes a large prophage island that is not predicted to encode a viable phage^3^, but whose transcription is up-regulated on cellulose and biomass (Supplementary Fig. 3). This burst of transcriptional initiation at viral genes could indicate prophage excision was triggered on cellulosic substrates, that is, by low carbon stress, or that viral proteins contribute to bacterial fitness^17^.

Sequences upstream of primary TSS generally contain the sigma-A-type consensus −35 and −10 hexamers recognized by RNA polymerase (RNAP) and associated elements that likely contribute to promoter function in this organism. An A-rich region upstream of the -35 hexamer (TTGACA) (Fig. 2h) resembles the ‘UP element' that stimulates transcription initiation by interacting with the RNAP alpha subunit^18^. Also, the Pribnow hexamer (TATAAT) has an upstream TG di-nucleotide (Fig. 2i), which enhances transcription in certain other bacteria^19^,^20^,^21^ by interacting with the RNAP sigma-A subunit^22^. In contrast, searching upstream of IntraS TSS identified an AT-rich stretch ∼10 bp upstream of the TSS lacking RNAP binding sites (Supplementary Fig. 4A), suggesting IntraS TSS often result from promiscuous initiation at AT-rich sequences. We observed IntraS TSS comprised that more than 50% of TSS (Fig. 2d), albeit with fewer reads per site than InterS TSS. dRNA-seq studies have rationalized similarly abundant intragenic TSS as resulting from incomplete TEX degradation^12^, but our data support these TSS bear 5′-PPP indicative of transcription initiation. IntraS TSS are preferentially found in the 5′ end of genes (Supplementary Fig. 4B), supporting they are under selective pressure and may have roles including expression of alternative protein isoforms or as mimicry molecules to sequester other RNA and ribonucleases from their mRNA targets^9^.

**Figure 3:**
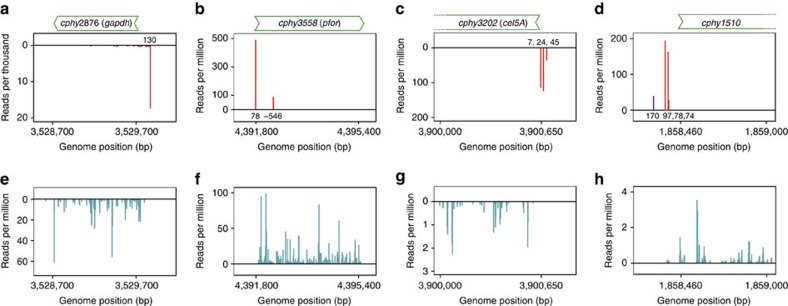
Capp-Switch reads start at specific genome positions corresponding to putative TSS. The number of reads starting at each genome position are shown for Capp-Switch (**a**–**d**) and RNA-seq (**e**–**h**). The *cphy2876 gapdh* gene (**a**,**e**) has a single TSS (glucose data shown). The *cphy3558 pfor* gene (**b**,**f**) has an upstream TSS and an intragenic sense TSS (glucose data shown). The *cphy3202 cel5A* cellulase gene (**c**,**g**) has three TSS during growth on cellulose. The *cphy1510* xylanase gene (**d**,**h**) is expressed from three TSS on xylan (red bars) and a single, upstream TSS on pectin (purple). Plots show the number of reads starting at each genome position with forward strand reads on the positive *Y* axis and reverse strand reads on the negative *Y* axis. Distance to the start codon is shown at the base of TSS peaks.

Capp-Switch reads (Fig. 3a–d) start at specific positions with respect to known genes showing TSS at single base resolution, whereas RNA-seq reads begin throughout genes (Fig. 3e–h). We observed four common TSS situations: genes with a single upstream TSS, genes with both upstream and intragenic TSS, genes with multiple TSS on a single substrate and genes with substrate-specific TSS. For example, the glyceraldehyde 3-phosphate dehydrogenase (*gadph*) gene is constitutively transcribed from a single TSS (Fig. 3a). The pyruvate ferredoxin oxidoreductase (*pfor*) gene is transcribed from a single, upstream TSS and another, weaker TSS in the coding sequence (Fig. 3b). The *cel5A* cellulase gene^23^ is simultaneously transcribed from multiple TSS on cellulose (Fig. 3c), as are other cellulases (Supplementary Fig. 5). CAZyme expression in *C. phytofermentans* is controlled by carbon source^24^,^25^ and our data supports their regulation involves multiple promoters. The *cphy1510* gene encoding the most active xylanase^5^ is transcribed from three TSS on xylan and a different, upstream TSS on pectin (Fig. 3d). Similarly, genes for other CAZymes including three cellulases, one other xylanase, four pectinases and two glycosyl transferases changed their primary TSS as a function of carbon source. We confirmed the positions of the primary TSS identified by Capp-Switch for *gadph*, *pfor* (IntraS and primary TSS), *cphy2243* and *cphy1510* (xylan and pectin) using 5′ RACE (Supplementary Fig. 6).

### Motifs associated with TSS clusters

**Figure 4:**
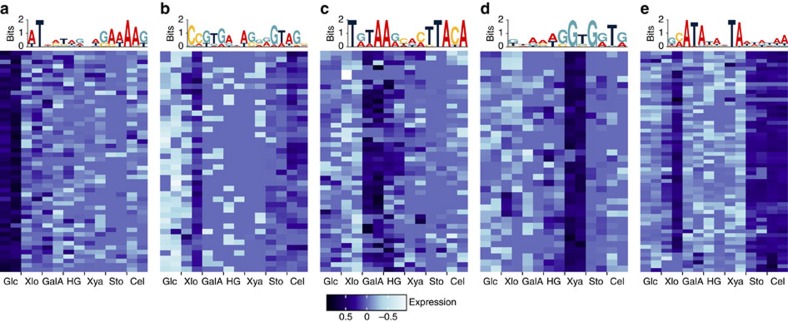
TSS in carbon source-specific clusters share DNA sequence motifs. TSS clusters differentially expressed on (**a**,**b**) glucose, (**c**) galacturonic acid and HG, (**d**) xylan and (**e**) stover and cellulose are shown along with their associated sequence motifs. Rows are expression of a TSS cluster member and columns are duplicate glucose (Glc), xylose (Xlo), galacturonic acid (GalA), homogalacturonan (HG), xylan (Xya), stover (Sto) and cellulose (Cel) cultures. Colours show TSS expression as log_2_-transformed read counts scaled to a median of zero for each TSS.

**Figure 5:**
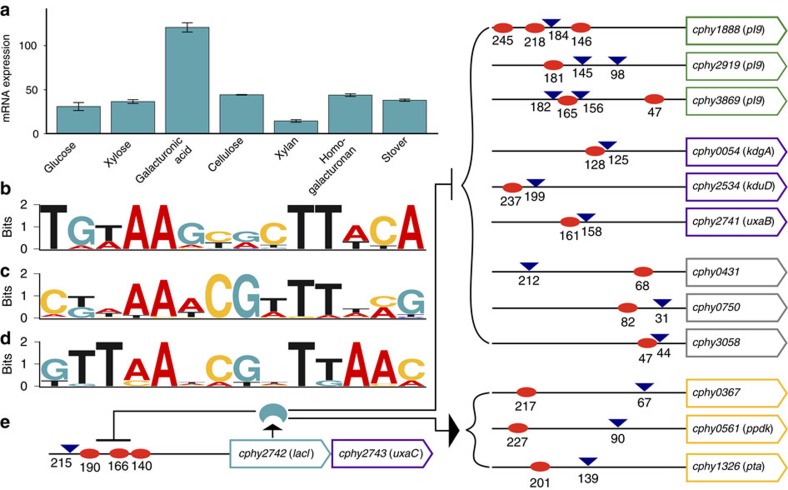
The role of the LacI/GalR regulator Cphy2742 in galacturonic acid and pectin metabolism. (**a**) Transcription of the LacI/GalR gene *cphy2742* is up-regulated on galacturonic acid relative to other carbon sources. Bars shows average RNA-seq RPKM of duplicate cultures; error bars are one s.d. (**b**–**d**) Upstream palindromes resembling *cre* operator sites found upstream of *C. phytofermentans* LacI/GalR genes and their orthologs from related genomes (**b**) *cphy2742* (motif *e*=1.1 × 10^−8^), (**c**) *cphy2467* (motif *e*=2.4 × 10^−8^) and (**d**) *cphy1883* (motif *e*=8.9 × 10^−2^). (**e**) Twelve genes have both TSS (blue triangles) and putative Cphy2742 operators (red ovals) including genes for pectin lyases (green), galacturonic acid metabolism (purple), general carbon metabolism (yellow) and other or unknown (grey). The distance from the translation start is shown for each site.

We clustered TSS based on expression across carbon sources and searched sequences surrounding TSS for overrepresented motifs (Supplementary Fig. 7; Supplementary Data 3), revealing TSS clusters that share motifs with potential regulatory functions (Fig. 4). For example, the TSS cluster up-regulated on galacturonic acid and homogalacturonan (HG) (Fig. 4c) has a palindromic motif resembling the *cre* operator (TGAAAGCGCTTTCA) bound by *B. subtilis* CcpA^26^,^27^, a LacI/GalR regulator of numerous carbon metabolism genes. LacI/GalR genes often have upstream copies of their operators to auto-repress transcription^28^, and we found three copies of the galacturonic acid cluster motif in the 5′ UTR of *cphy2742*, a LacI/GalR gene specifically up-regulated on galacturonic acid (Fig. 5a). Further, three of the six LacI/GalR genes with detected primary TSS have upstream variants of the *cre* operator that are conserved in their orthologs from related species (Fig. 5b–d), leading us to propose *C*. *phytofermentans* LacI/GalR regulators recognize related, but distinct, operators to control separate regulons. Supportingly, the putative Cphy2742 operator (Fig. 5b) is upstream of 22 genes in the *C. phytofermentans* genome (Supplementary Table 1) including 3 CAZymes (PL9 pectin lyases) that degrade HG to galacturonic acid^5^ and transcription units containing all genes needed to assimilate galacturonic acid^29^ (Supplementary Fig. 8).

The putative Cphy2742 operator sites are co-located with or downstream of TSS for HG degradation and galacturonic acid metabolism genes (Fig. 5e), supporting Cphy2742 binds these sites to block transcription. Transcription of the *pl9* genes *cphy2919* and *cphy3869* switches to upstream primary TSS on galacturonic acid relative to HG, but all TSS are close enough to be potentially regulated by Cphy2742 operators. The *pta-ackA* (*cphy1326-7*) acetate synthesis operon also has a Cphy2742 operator and both *pta-ackA* expression and acetate formation are elevated on galacturonic acid (Supplementary Fig. 9). While *B. subtilis* CcpA represses most of its targets, it activates *pta* and *ackA* transcription^30^,^31^ by binding upstream of their promoters^32^. The Cphy2742 operator is also upstream of the *pta* gene TSS, suggesting Cphy2742 may similarly activate transcription of the *pta-ackA* operon as well as the glycolytic gene *ppdK* and the hydrolase gene *cphy0367*. Collectively, we propose Cphy2742 represses a comprehensive set of pectin fermentation genes by binding a conserved palindrome at or downstream of their TSS to block transcription. In response to a galacturonic acid-based signal, Cphy2742 de-represses itself and its targets, and may activate transcription of acetate synthesis and other aspects of carbon metabolism by binding upstream of TSS.

### Antisense and novel transcripts

**Figure 6:**
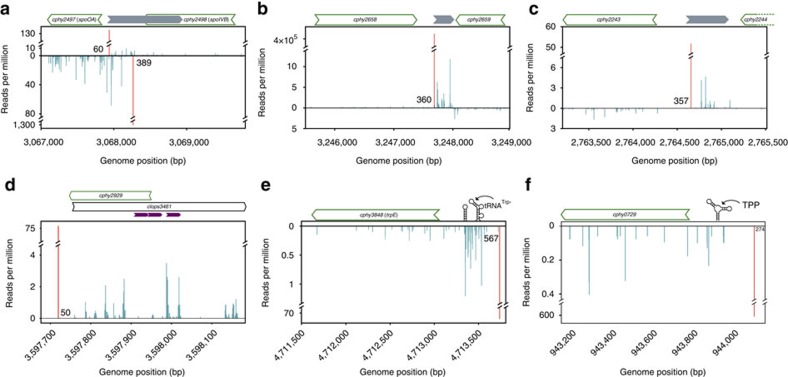
TSS show genome features. (**a**) The *cphy2497 spoOA* gene has both a primary TSS and an antisense TSS in the 5′ UTR (grey arrow) that were observed on all sugars (glucose data shown). (**b**) A novel transcription unit (grey arrow) is up-regulated to be the most highly expressed TSS on biomass. (**c**) Induction of a transcription unit (grey arrow) upstream of the ABC transporter *cphy2243* is associated with repression of the transporter. This TSS was observed on all substrates except glucose (cellulose data shown). (**d**) A primary TSS, RNA-seq reads, and three in-frame peptides expressed on cellulose support the MaGe-predicted *clops3461* gene rather than the annotated *cphy2929* gene. Positions of peptides detected by mass spectrometry (purple) are shown. (**e**) The *trpE* (*cphy3848*) gene has an upstream T-box that terminates transcription in the 5′ UTR during log-phase growth on glucose. (**f**) The thiamine transporter (*cphy0729*) has an extended 5′ UTR containing a TPP-binding riboswitch. All plots show the number of reads starting at each genome position for RNA-seq (blue) and Capp-Switch (red). Numbers at base of TSS peaks are distances to start codons of (**a**) *cphy2497*, (**c**) *cphy2243*, (**d**) *clops3461,* (**e**) *cphy3848*, (**f**) *cphy0729* and (**b**) the *cphy2659* stop codon.

Recent studies found 30–40% of TSS are antisense in other bacteria^8^,^9^,^13^. However, antisense transcription appears rare in *C. phytofermentans:* <1% of TSS were antisense either between (InterA) or within genes (IntraA) (Fig. 2d). To further investigate whether diffuse antisense transcription was underestimated by our TSS thresholds, we classified all mapped read starts, including those not meeting TSS thresholds. Even then, InterA and IntraA classes together comprise <4% reads. This dearth of antisense transcription may relate to the early evolutionary divergence of the Clostridiales^33^. Alternatively, we would not detect antisense transcripts that were processed to remove 5′-PPP or that are below the 200 bp size threshold of our cDNA libraries, but studies in other bacteria using larger size thresholds found antisense TSS in ∼35% of genes^10^. While comparatively rare, antisense transcription appears to have important cellular functions. For example, we observed an antisense TSS in the 5′ UTR of the sporulation regulator *spoOA* (*cphy2497*) that also opposes transcription of the *spoIVB* peptidase (*cphy2498*) (Fig. 6a). This TSS was expressed on all sugars, but not polysaccharides, supporting antisense transcription has a role in repressing sporulation during log growth in sugar-replete conditions.

TSS reveal novel transcriptional features such as a TU downstream of the glycoside hydrolase *cphy2658* that is up-regulated to have the strongest initiation site in the genome on cellulose and corn stover (Fig. 6b). This region contains a hypothetical open-reading frame (ORF) in the MaGe annotation (*clops3132*) that has no similar sequences in Genbank, but the ORF lacks an ribosome binding site (RBS), and we did not detect any expressed peptides from this region by mass spectrometry, suggesting it is a non-coding RNA. The most highly expressed ABC transporter on glucose is a putative operon (*cphy2241-3*) with a single TSS (Supplementary Fig. 5C,F). On all other carbon sources, we observed repression of *cphy2241-3* along with appearance of an upstream, antisense TU (Fig. 6c) that has no mapped peptides or predicted ORF. Non-coding RNA are often associated with ABC transporters in clostridia^34^, and they may also regulate ABC transport in this organism.

The *C. phytofermentans* genome may encode significantly more genes than in the NCBI Genbank annotation. Classifying TSS using the MaGe annotation showed 735 (7%) TSS map to MaGe-specific *clops* genes of unknown function (Supplementary Data 4), including 64 *clops* genes with InterS TSS. We examined which of these novel TU encode proteins by mapping *C. phytofermentans* MS/MS peptide spectra to the genome translated in all frames, identifying peptides outside the predicted proteome in 21 InterS, 13 IntraS, 5 InterA and 25 IntraA regions (Supplementary Data 5). The combination of TSS and expressed peptides supports ORFs with N-terminal extensions such as *cphy0891* (Supplementary Fig. 10A) and the existence of novel ORFs. For example, *clops3461*, which overlaps with *cphy2929* on the opposite strand (Fig. 6d), and an antisense overlapping ORF in *cphy1953* encoding the ComEA competence protein (Supplementary Fig. 10B).

TSS also show mechanisms of RNA-mediated gene regulation. Comparative genomics with other clostridia detected a putative T-box upstream of the *C. phytofermentans trp* operon^34^. In low tryptophan conditions, the T-box promotes antitermination of the *trp* operon by base pairing with uncharged tRNA^trp^ (ref. 35). We observed transcription halted abruptly in the 5′ UTR of the *trp* operon in glucose cultures (Fig. 6e), consistent with T-box-mediated repression. In cellulose cultures, antitermination in the T-box enabled *trp* operon mRNA expression, potentially enabling translation of the trytophan-rich carbohydrate binding modules in cellulases and other CAZymes. TSS also support riboswitches associated with genes for metabolism of flavin mononucleotide (FMN), cobalamin, thiamine pyrophosphate (TPP) and lysine (Supplementary Data 6). For example, *C. phytofermentans* is auxotrophic for thiamine, which it uptakes by a thiamine transporter, Cphy0729 (ref. 36). The *cphy0729* gene has a single, constitutive TSS with an extended 5′ UTR containing a putative TPP-sensing riboswitch (Fig. 6f) that could regulate transporter expression in response to intracellular TPP levels^37^.

## Discussion

The strategy presented here to quantify condition-specific changes in transcription initiation by Capp-Switch sequencing could be generally applied to dissect the regulation of complex bacterial phenotypes. In this study, we explored the transcriptional programme enabling *C. phytofermentans* to ferment the cellulosic, hemicellulosic and pectic components of plant biomass. We found that growth on these different carbon sources entailed widespread TSS changes, including use of substrate-specific TSS for genes encoding biomass-degrading enzymes such as cellulases, xylanases and pectinases. Substrate-specific TSS could enable tuning of expression by changing promoters or the regulatory properties (that is, binding sites or secondary structure) of the 5′ UTR. We observed that genes encoding cellulases and other enzymes are simultaneously expressed from more than one TSS. Multiple regulators may control transcription of these genes, reflecting the numerous transcription factors encoded by this organism (Supplementary Data 7). Genes for biomass-degrading enzymes in other Clostridiales are regulated by various transcription factors including a two-component system for hemicellulases^38^, a LacI/GalR protein for β-1-3 glucanases^39^ and alternative sigma factors for cellulases^40^. We defined TSS clusters that were differentially expressed on specific carbon sources and used them to guide the discovery of sequence motifs with potential regulatory function, leading us to identify the LacI/GalR Cphy2742 as a putative regulator of pectin metabolism. Combining TSS mapping with motif searching could be broadly applied to LacI/GalR regulators and other types of transcription factors. For example, each of the 4 TetR regulators for which we detected TSS also have conserved, TSS-associated palindromes that resemble operator sites (Supplementary Fig. 11).

We also gained insight into regulatory mechanisms such as antisense transcription, leaderless transcription and non-coding RNA. We observed that antisense and leaderless transcription are much rarer than reported in other bacteria and it will be interesting to see if they are similarly uncommon in closely-related bacteria. We also show that integration of Capp-Switch TSS mapping with RNA-seq and proteomics enables discovery of novel transcription units and protein-encoding genes. Transcription initiation is a complex and important component of gene regulation for which most of the underlying mechanisms in *C. phytofermentans* are yet unknown. Further, these results illustrate how little we know about gene regulation in plant-fermenting clostridia, a group of bacteria with important roles in soil and gut microbiomes that have significant potential to serve as biocatalysts for industrial transformation of plant biomass.

## Methods

### Bacterial cultivation

*C. phytofermentans* ISDg (ATCC 700394) was cultured anaerobically at 30 °C in GS2 medium^41^ containing 5 g l^−1^ of either D-(+)-glucose (Sigma G5767), D-(+)-xylose (Sigma X3877), D-galacturonic acid sodium salt (Sigma 73960), regenerated amorphous cellulose (RAC) from Avicel PH-101 (Sigma 11365), birchwood xylan (Sigma X0502), apple pectin (HG) (Sigma P8471) or raw corn stover (Qteros Inc) cut in 0.5 × 3.0 cm strips. RAC was prepared by phosphoric acid treatment^42^. Duplicate cultures were sampled in mid-log phase or after 2 days (RAC) or 3 days (stover). Fermentation products were quantified by HPLC^43^.

### Capp-Switch library preparation

Total RNA was extracted from duplicate cultures for each treatment using TRI reagent (Sigma 93289) and treated with Turbo DNase (Ambion AM2238) at 0.2 U μg^−1^ RNA for 30 min at 37 °C. RNA was purified by Zymo Concentrator-5 (Zymo Research R1015) (>200 bp capture) into 15 μl water. RNA was 5′ capped using VCE (NEB M2080) at 3 U μg^−1^ RNA with 0.1 mM SAM and 0.5 mM 3′ biotin-GTP (NEB N0760) for 30 min at 37 °C and purified by Zymo Concentrator-5 (>200 bp capture) with two additional washes into 45 μl water. RNA was fragmented for 30 s at 94 °C using NEBNext Magnesium-based RNA fragmentation buffer (NEB E6101) and purified by Zymo Concentrator-5 (total RNA capture) into 100 μl water. Streptavidin magnetic beads (NEB S1421S) were pre-washed twice with low-salt buffer (10 mM Tris, 50 mM NaCl, 1 mM EDTA), twice with binding buffer (10 mM Tris, 500 mM NaCl, 1 mM EDTA) and resuspended at 4 mg ml^−1^ beads in binding buffer. Capped RNA fragments were bound to streptavidin beads for 20 min at room temperature and magnetically separated from other RNA by washing twice with binding buffer and twice with low-salt buffer to elute non-bound RNA. Beads were washed once with 1 mM Tris–HCl pH 7.5 and resuspended in 1 mM Tris–HCl pH 7.5.

RNA was converted to single-strand cDNA by SMARTscribe MMLV reverse transcriptase (Clontech 634836) at 10 U μl^−1^ with 2.5 mM DTT, 1 mM dNTP, 1.2 μM SMARTer stranded oligo and 0.6 μM SMART stranded N6 primer (Clontech 634836) by incubating 90 min at 42 °C and 10 min at 70 °C. Beads were collected and the supernatant was combined with the liquid fraction after the beads were washed with 30 μl 1 mM Tris pH 7.5. The cDNA was twice purified using 1 volume of solid phase reversible immobilization (SPRI) beads (Beckman Coulter A63880). cDNA was left on beads after the second purification and double-stranded cDNA was synthesized by 18 cycles PCR using SeqAmp DNA polymerase (Clontech 638504) with 0.25 μM primers (Universal Forward PCR primer and indexed Reverse PCR primer) and then SPRI purified with 1 volume of beads. DNA was sequenced on Illumina MiSeq with 150 bp paired-end reads chemistry.

### TSS identification and classification

Sequencing reads were quality filtered^44^ and the 3 bp MMLV reverse transcriptase 3′ non-template extension was removed from the 5′ end of forward (R1) reads. Reads were mapped to the *C. phytofermentans* ISDg genome (NCBI NC_010001.1) using Bowtie 2 (version 2.2.4)^45^. Alignments showed 87–98% of reads mapped to unique positions in the *C. phytofermentans* genome, yielding between 0.4 million (corn stover) and 3.4 million (glucose) reads per culture (Supplementary Table 2). TSS were identified using R1 reads by calculating the number of reads starting at each genomic position, clustering read counts within a 5 bp sliding window, and retaining the position with the greatest number of reads. TSS were defined as genome positions with greater than 10 read starts per million reads in both duplicate cultures. Capp-switch TSS were confirmed by 5′ RACE (Sigma 03353621001) using primers in Supplementary Table 3 to amplify PCR products, which were resolved by electrophoresis, excised and sequenced.

Genes in the NCBI and MicroScope (MaGe) annotations^46^ were used to divide TSS into four categories: InterS (intergenic TSS with downstream gene in same orientation), InterA (intergenic TSS with downstream gene opposite orientation), IntraS (intragenic TSS in gene with same orientation) or IntraA (intragenic TSS in gene with opposite orientation). The InterS TSS with the most reads for each gene was defined as the primary TSS. Capp-Switch results were compared with strand-specific (dUTP) RNA-seq of *C. phytofermentans* grown in the same culture conditions^5^. RNA-seq gene expression was calculated as RPKM using the Bioconductor^47^ package ‘easyRNASeq' and differential expression was defined as a DESeq^48^ (version 1.22.1) *P*-value <0.05 adjusted for multiple testing of the 3,902 genes in *C. phytofermentans* genome by Bonferroni correction. Peptides corresponding to novel ORFs were identified by mapping peptide MS/MS spectra from glucose, xylan and cellulose cultures^4^ to the genome translated in all six frames. Peptides were identified from spectra using SEQUEST and filtered to a 5% false discovery rate using a target-decoy approach^49^,^50^ including a target database and a decoy of the reversed sequences.

### Motif analysis

Sequence motifs were identified using MEME^51^ with a background model of di-nucleotide frequencies in the *C. phytofermentans* genome. Searches for RNA polymerase binding site motifs included positions 25–50 bp (−35 motif) and 5–20 bp (−10 motif) upstream of all primary TSS expressed on the three sugars and polysaccharides. The top palindromic motifs associated with LacI/GalR and TetR regulators were found by searching sequences from −250 (upstream) to +50 bp (downstream) relative to the start codon of *C. phytofermentans* genes and their putative orthologs from related genomes identified by top reciprocal BLAST searches (Supplementary Table 4). These motifs were used for genome-wide scans from −250 to +50 bp within all *C. phytofermentans* genes using MAST^52^. To cluster TSS by expression, the 1,188 TSS with at least a 30-fold change in read counts between two conditions were log_2_-transformed and each TSS was normalized to have a median value of 0 across conditions and scaled so the sum of the squared expression levels is 1. TSS were separated into 24 clusters by *K*-means using the city-block similarity metric. Significant motifs (*e*<0.001) associated with individual *K*-means clusters were identified by searching −100 to +10 bp with respect to each TSS.

### Data availability

The authors confirm that all data underlying the findings are fully available without restriction. RNA sequencing files in FASTQ format are available in the European Nucleotide Archive under study accession PRJEB13063.

## Additional information

**How to cite this article**: Boutard, M. *et al*. Global repositioning of transcription start sites in a plant-fermenting bacterium. *Nat. Commun.* **7**, 13783 doi: 10.1038/ncomms13783 (2016).

**Publisher's note:** Springer Nature remains neutral with regard to jurisdictional claims in published maps and institutional affiliations.

## Supplementary Material

Supplementary Information Supplementary Figures and Supplementary Tables

Supplementary Data 1 Classification of 9,457 TSS based on the *C. phytofermentans* ISDg NCBI annotation (NC_010001.1). TSS were categorized as either InterS (3,519 TSS), IntraS (5,721 TSS), InterA (67 TSS), or IntraA (150 TSS). Three highly-expressed TSS associated with hypothetical genes not in the NCBI annotation are included: *cphy2658a* (WP_041703693), *cphy0846a* (WP_049762298), and *cphy0846b* (WP_041703193). Table includes TSS position, strand, class, downstream gene, start position of downstream gene, distance to gene, gene annotation, and average reads between duplicate cultures normalized to one million total reads per carbon source. Distances are from TSS to start for InterS, IntraS, IntraA and from TSS to gene stop for InterA.

Supplementary Data 2 Computational prediction of 1,829 *C. phytofermentans* operons that include 3,630 genes using MicrobesOnline. Table rows show transcription direction and gene members for each operon

Supplementary Data 3 TSS members of K-means clusters. Table includes TSS position, strand, and cluster name for clusters shown in Supplementary Fig 7

Supplementary Data 4 Classification of 9,457 TSS based on the MaGe annotation. TSS were categorized as either InterS (3,266 TSS), IntraS (5,874 TSS), InterA (55 TSS), or IntraA (262 TSS). Table includes TSS position, strand, class, downstream gene, start position of downstream gene, distance to gene, gene annotation, and average reads between duplicate cultures normalized to one million total reads per carbon source. Distances are from TSS to start codon for InterS, IntraS, IntraA and TSS to stop codon for InterA.

Supplementary Data 5 Peptides detected by mass spectrometry mapping outside of predicted proteome. Table shows peptide sequence, translation frame, genome location, number of observances, surrounding genes, and proposed annotation. Peptides were categorized similar to TSS as either intergenic sense, intergenic antisense, intragenic sense, or intragenic antisense

Supplementary Data 6 TSS support expression of non-coding RNA. *C. phytofermentans* (RFAM taxon CP000885.1) encodes 134 non-coding RNA according to the RFAM 12.0 database. Page 1: TSS associated with non-coding RNA: riboswitches, RNase, sRNA sau-50, SRP subunits, and group II introns. Page 2: Translation RNAs predicted by RFAM including 8 rRNA copies (5S, 16S, 23S), a 6S RNA, 61 tRNA, a tmRNA, 15 T-box leaders, and an L10 leader

Supplementary Data 7 *C. phytofermentans* transcription regulator proteins in the InterPro database. Table shows NCBI gene number, Uniprot ID, predicted function, protein length, and InterPro classifications. Pages 1 shows all 309 transcription factors and subsequent pages are classes with more than 10 members: two-component regulators (66), AraC (61), Sigma factors (26), LacI (25), XRE (22), GntR (20), TetR (19), MerR (14), LysR (12), MarR (12).

## Figures and Tables

**Figure 1:**
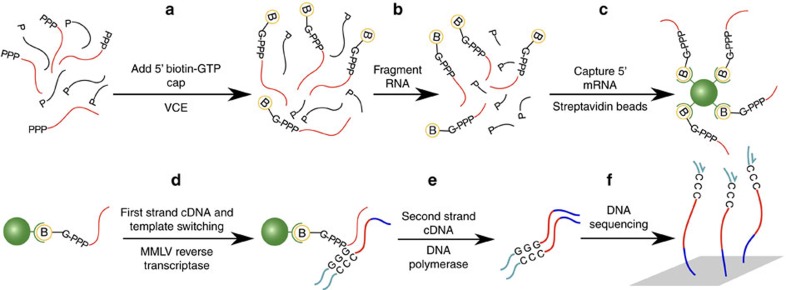
Overview of the Capp-Switch sequencing approach. Capp-Switch includes (**a**–**c**) capture of 5′ mRNA fragments and (**d**–**f**) cDNA synthesis and sequencing. (**a**) The mRNA 5′ triphosphate is capped with biotin-GTP by VCE. (**b**) RNA is fragmented and (**c**) the capped 5′ mRNA fragments are captured on streptavidin magnetic beads and separated from other RNA. (**d**) The 5′ mRNA fragments are reverse transcribed to single-stranded cDNA using MMLV reverse transcriptase. An oligonucleotide hybridizes to the 3′ overhang and the complementary sequence is synthesized by the MMLV template-switching activity. (**e**) Double-stranded cDNA is synthesized using primers that hybridize to the single-stranded cDNA termini. (**f**) The cDNA is sequenced on a high-throughput platform.
