# Supplementary material for: Global repositioning of transcription start sites in a plant-fermenting bacterium
Source: Nat Commun. 2016 Dec 16;7:13783. doi: 10.1038/ncomms13783 (PMC5171806; doi:10.1038/ncomms13783)
Supplement: Supplementary Information — Supplementary Figures and Supplementary Tables [file ncomms13783-s1.pdf]

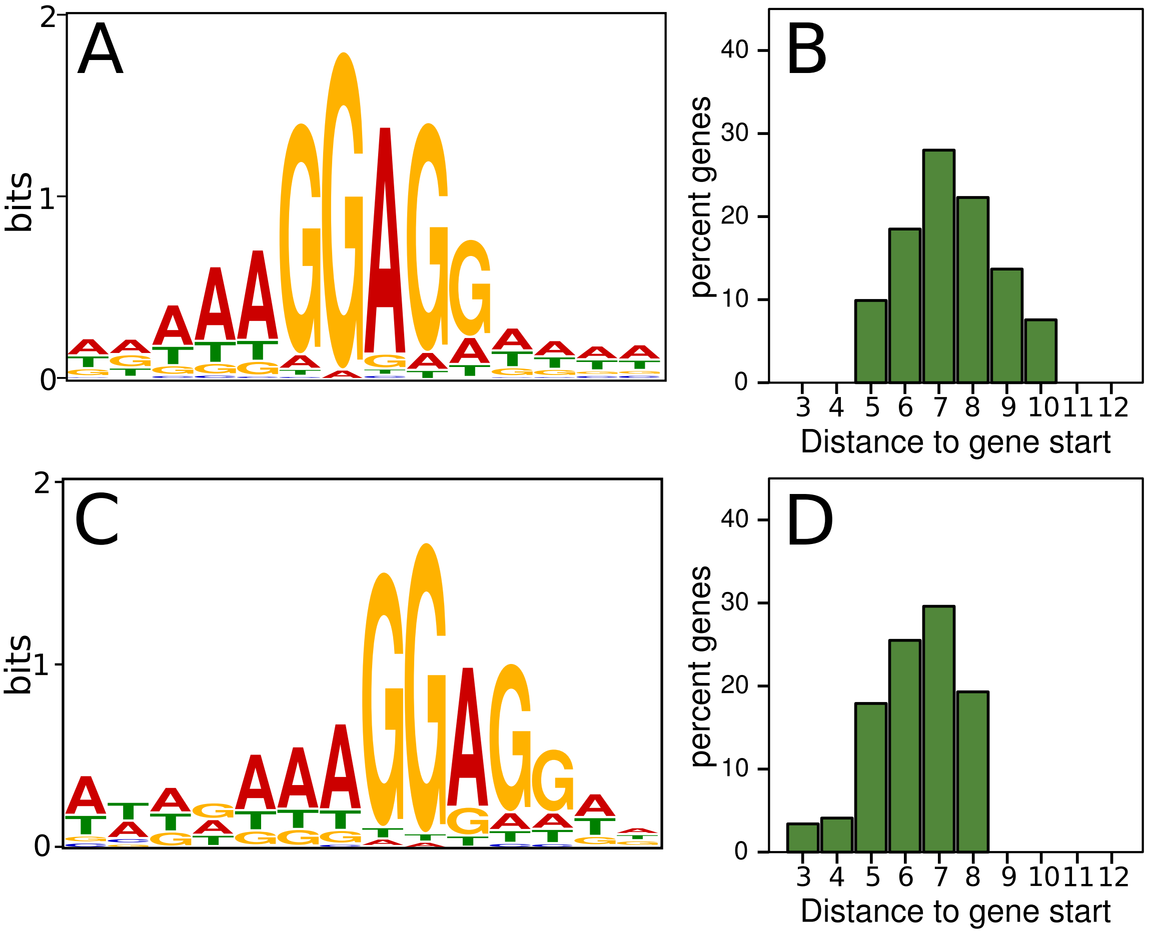

Supplementary Fig 1 Genes with leaderless transcripts have ribosome-binding motifs (RBS) similar to other genes. **A** *C. phytofermentans* genes encoding highly-expressed proteins have a classic RBS (5'-AGGAGG-3'). The 20 bp upstream of the 500 most highly-expressed proteins on glucose based on APEX values from mass spectrometry-based proteomics were searched using MEME and the top motif (e-value 4.6×10^{-173}, 475 sites) is shown. **B** The core AGGAGG motif is separated from the start codon by 7.3 +/- 1.38 bp for these 500 genes. Analysis of the 500 most highly expressed proteins on cellulose gave an indistinguishable RBS motif (motif e-value 3.2×10^{-734}, 465 sites) with similar start codon distances. **C** Among 3,540 InterS TSS identified by Capp-Switch sequencing, 173 TSS (4%) associated with 152 genes have TSS 5 bp or fewer from the start codon (leaderless). The 20 bp upstream of these 152 genes were searched using MEME and the top motif (e-value 2.4×10^{-167}, 145 sites) also resembles a ribosome-binding site. **D** The core AGGAGG motif for these 152 genes is closer to the start codon (6.3 +/-1.3 bp).

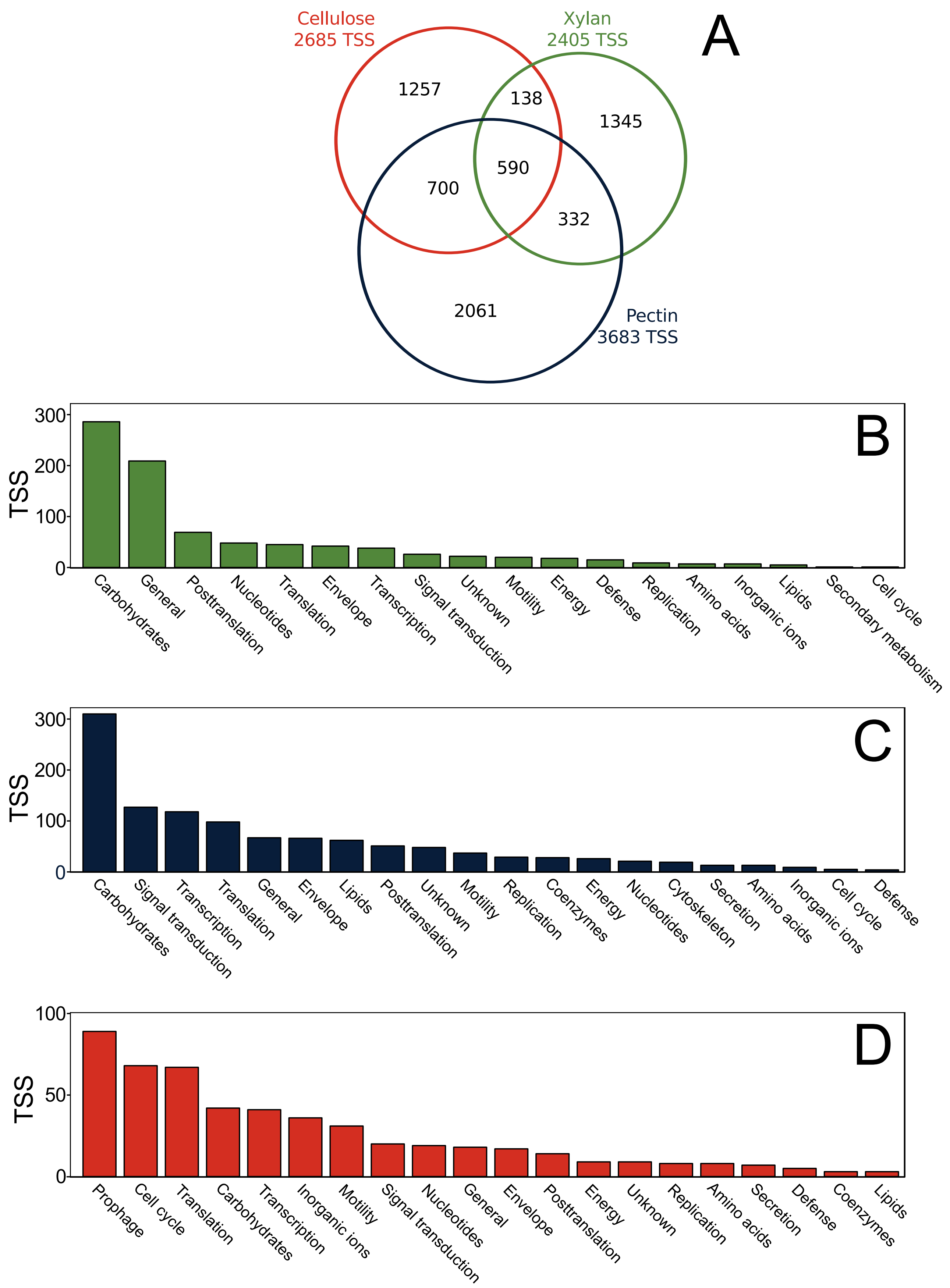

Supplementary Fig 2 Comparison of TSS during growth on 3 polysaccharides: cellulose, xylan, and pectin. **A** Venn diagram showing overlap of TSS identified on each substrate. Number of TSS specific to **B** xylan, **C** pectin, or **D** cellulose with associated genes divided into Clusters of Orthologous Genes (COG) functional categories.

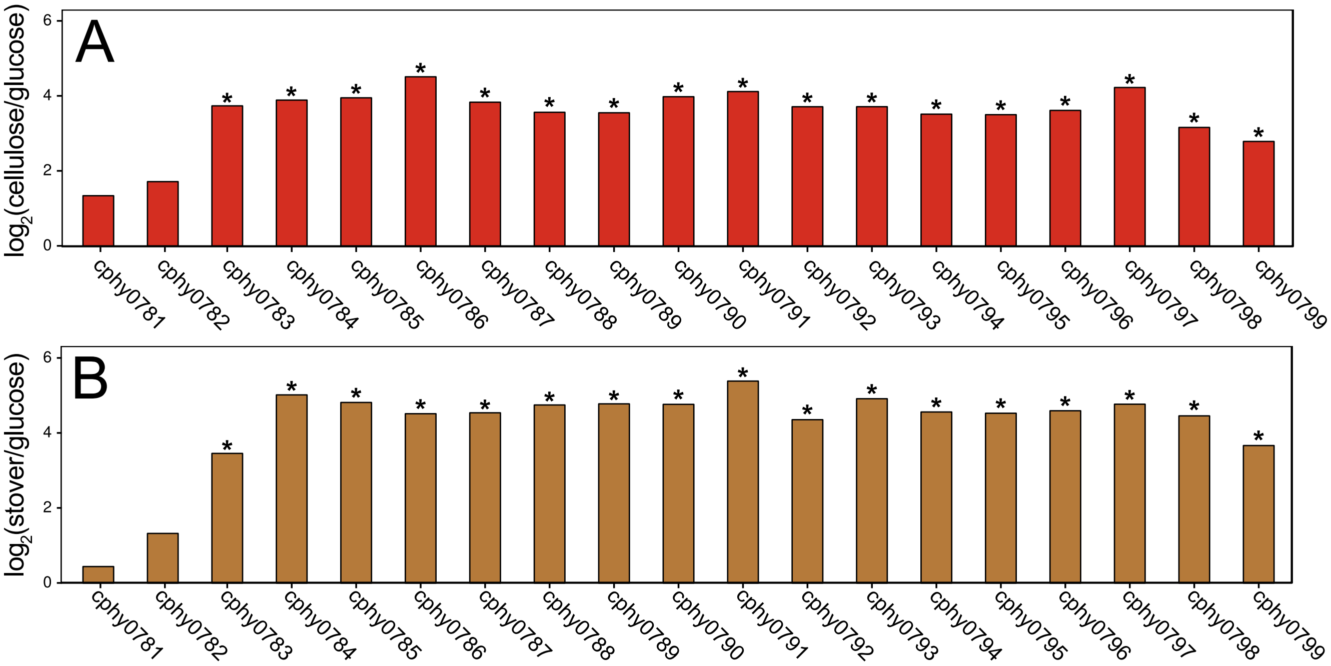

Supplementary Fig 3 *C. phytofermentans* genes in the prophage island (*cphy0781-cphy0799*) are up-regulated on **A** cellulose and **B** biomass (corn stover). The mRNA expression levels are RNA-seq $\log_2$-transformed ratios of RPKM values on cellulose or corn stover relative to glucose. Asterisks show significant up-regulation. In total, 250 genes were up-regulated on cellulose and 294 genes on stover across the genome.

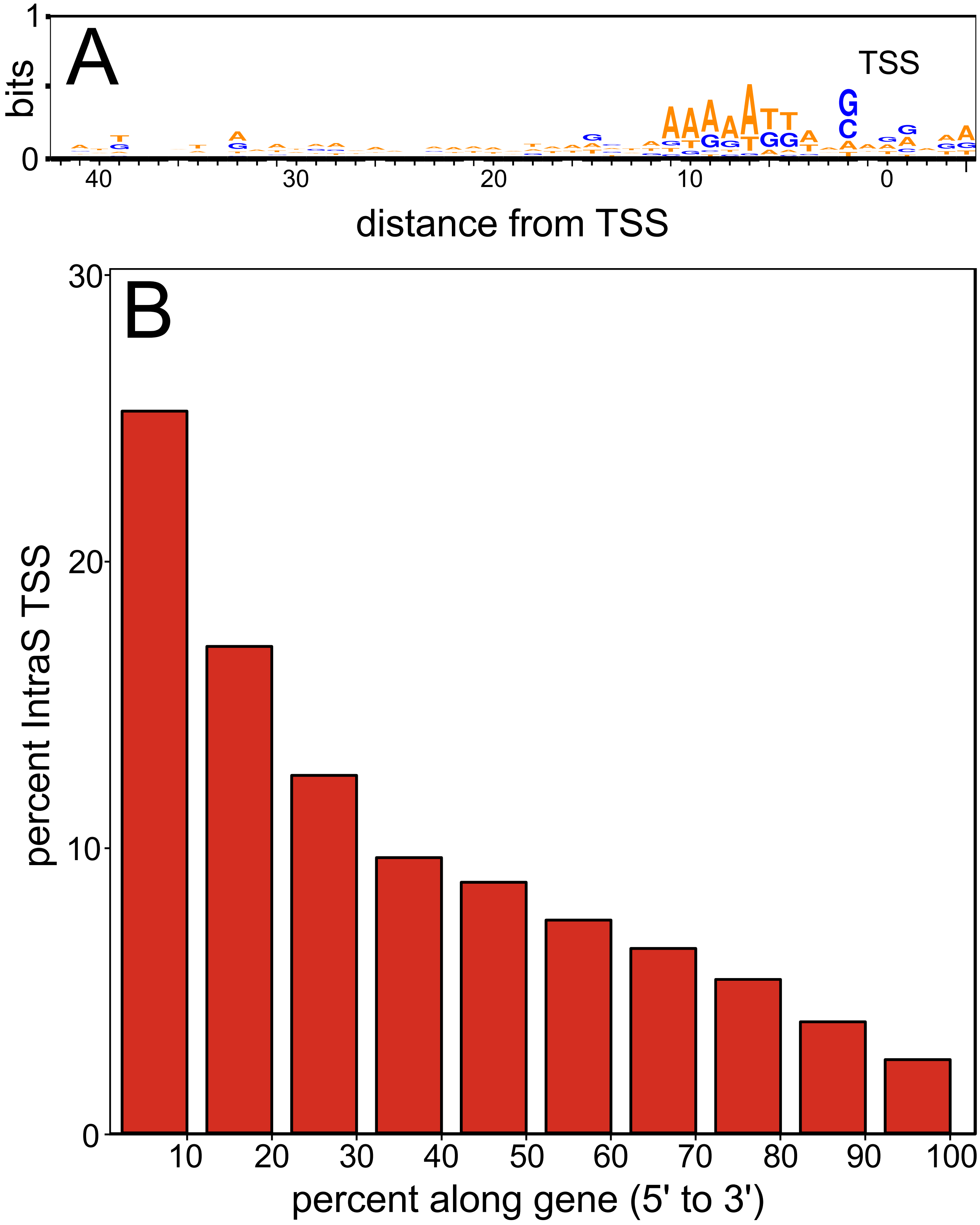

Supplementary Fig 4 A Sequences upstream of conserved IntraS TSS have an AT-rich stretch approximately 10 bp upstream from the TSS, but lack -10 and -35 RNA polymerase binding motifs found upstream of InterS TSS. Alignment shows sequences surrounding the 82 IntraS TSS meeting the following criteria: TSS is expressed on all three sugars, TSS has the most cumulative reads on sugars for that gene, and the associated gene has an annotated function. **B** Intragenic sense (IntraS) TSS are preferentially located in the 5' end of genes. Plot shows the percentages of IntraS located along genes from 5' to 3'.

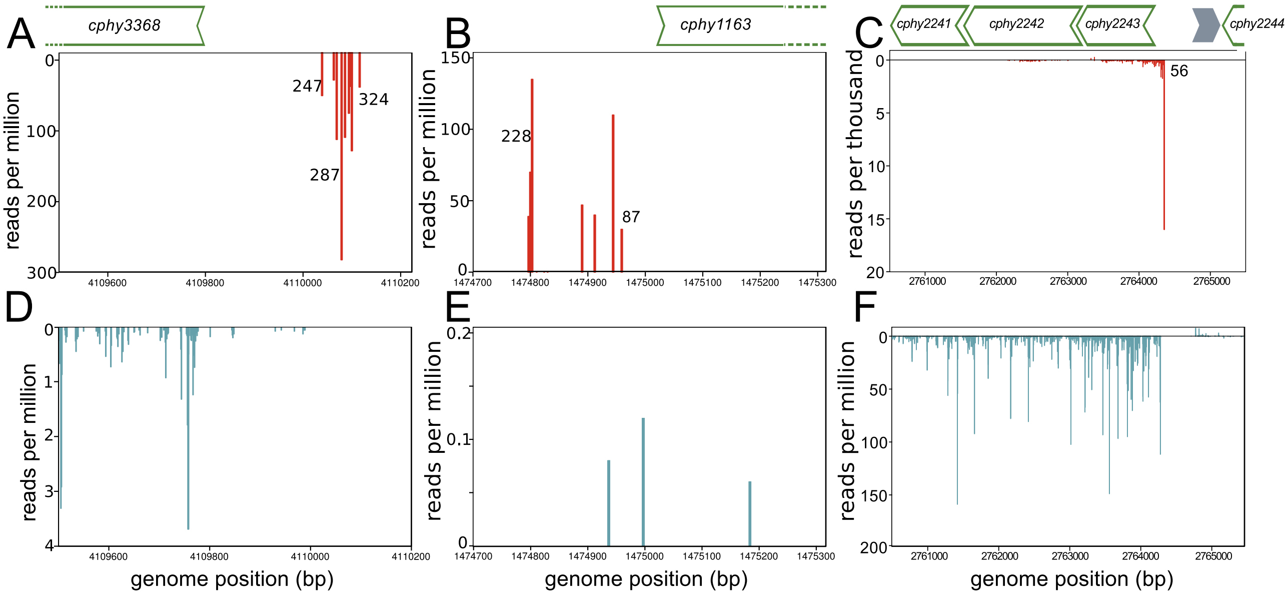

Supplementary Fig 5 Transcription of cellulase-encoding genes *cphy3368* (**A**, **D**), *cphy1163* (**B**, **E**), and the ABC transporter genes *cphy2241-3* (**C**,**F**). The number of reads starting at each genomic position is shown for Capp-Switch sequencing (**A**-**C**) and RNA-seq (**D**-**F**) and the distance to the start codon is shown for select TSS. Genomic positions of annotated genes and the transcription unit opposing the ABC transport operon (gray arrow) are shown above the plots.

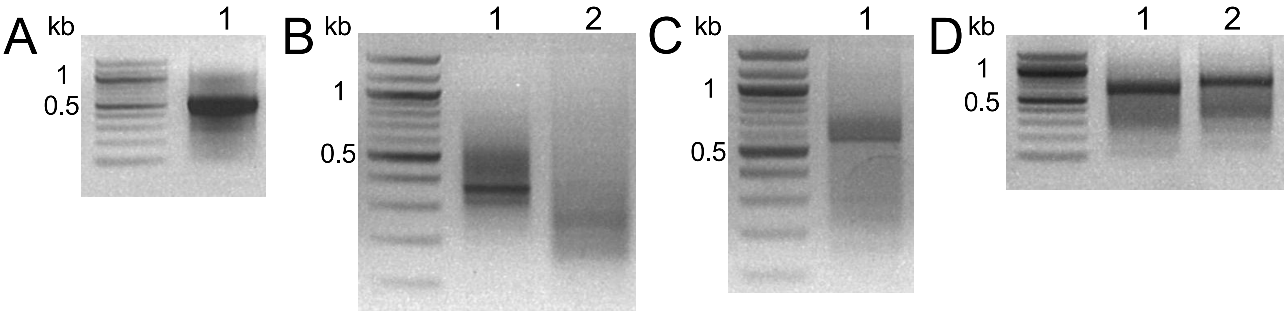

Supplementary Fig 6 Mapping transcript ends by 5’ RACE confirms expected start positions based on Capp-Switch TSS. **A** Lane 1: *cphy2876* (*gadph*) expected size 548 bp. **B** Lane 1: *cphy3558* (*pfor*) primary TSS expected size 338 bp. Lane 2: *cphy3558* (*pfor*) IntraS TSS expected size 274 bp. **C** *cphy2243* expected size 586 bp. **D** Lane 1: *cphy1510* xylan expected size 651 bp. Lane 2: cphy1510 pectin expected size 724 bp for primary TSS. Bands were excised and sequenced to confirm TSS positions.

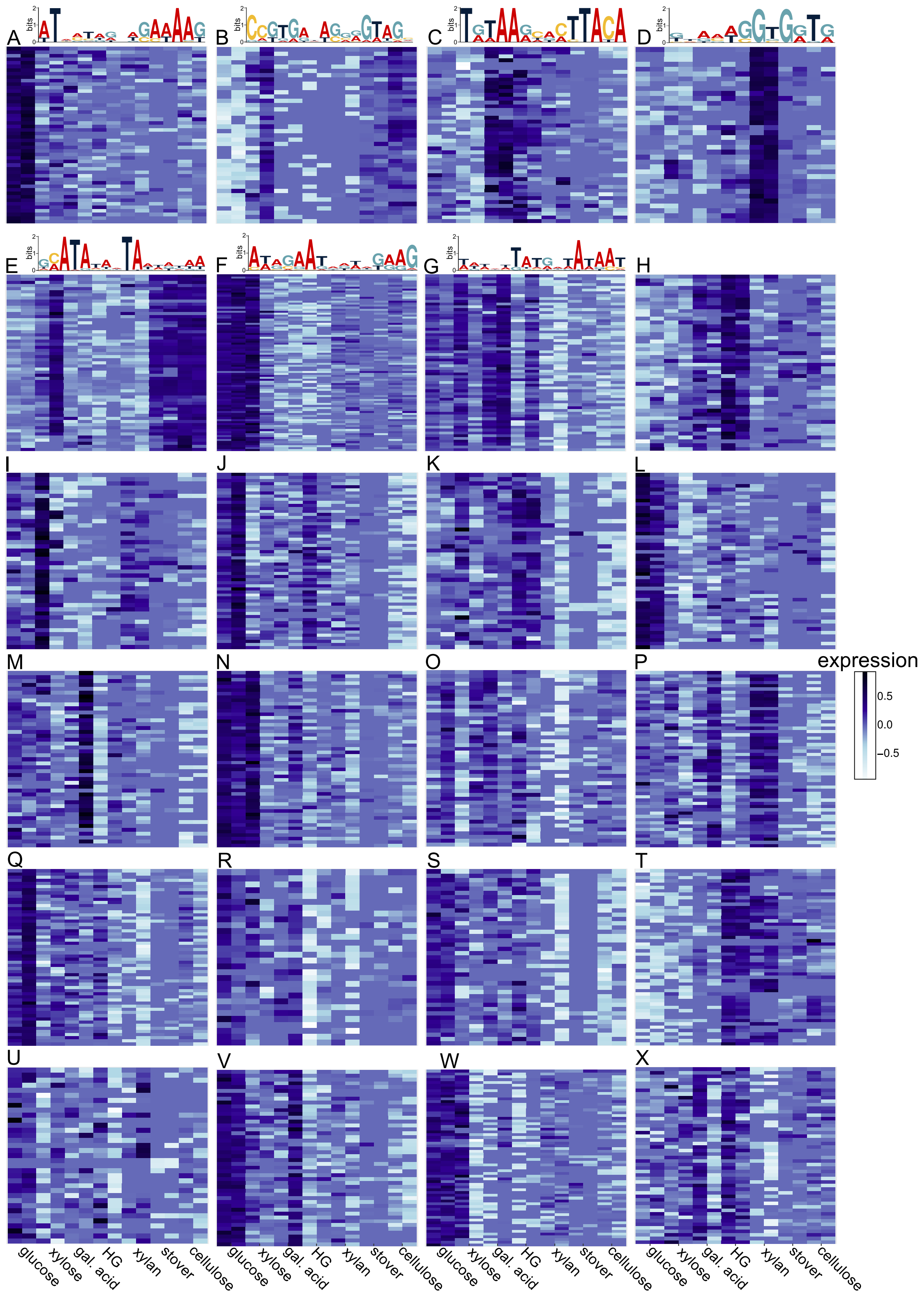

Supplementary Fig 7 K-means TSS clusters and associated sequence motifs. The 1,188 TSS with at least a 30-fold change between at least two conditions were separated into 24 clusters by K-means. Colors show TSS expression as log2-transformed read counts scaled to a median of zero for each TSS. Significant motifs ($e<0.001$) were found in 7 clusters by searching from 100 bp upstream to 10 bp downstream of each TSS. Motifs are shown above their associated clusters. Clusters A-E are shown in Fig 4.

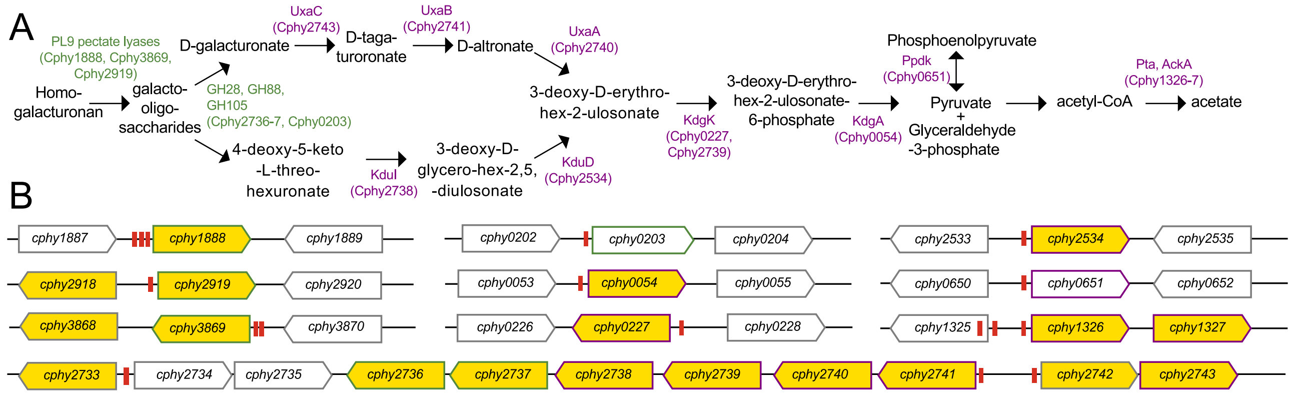

Supplementary Fig 8 A Proposed *C. phytofermentans* pathway for fermentation of homogalacturonan to acetate. **B** Genomic organization shows all genes in the pathway are in transcription units with upstream, putative Cphy2742 operator sites (red rectangles). Colors: green is homogalacturonan degradation, purple is galacturonic acid fermentation, yellow-filled genes are greater than 2-fold up-regulated on galacturonic acid relative to glucose measured as RPKM by RNA-seq.

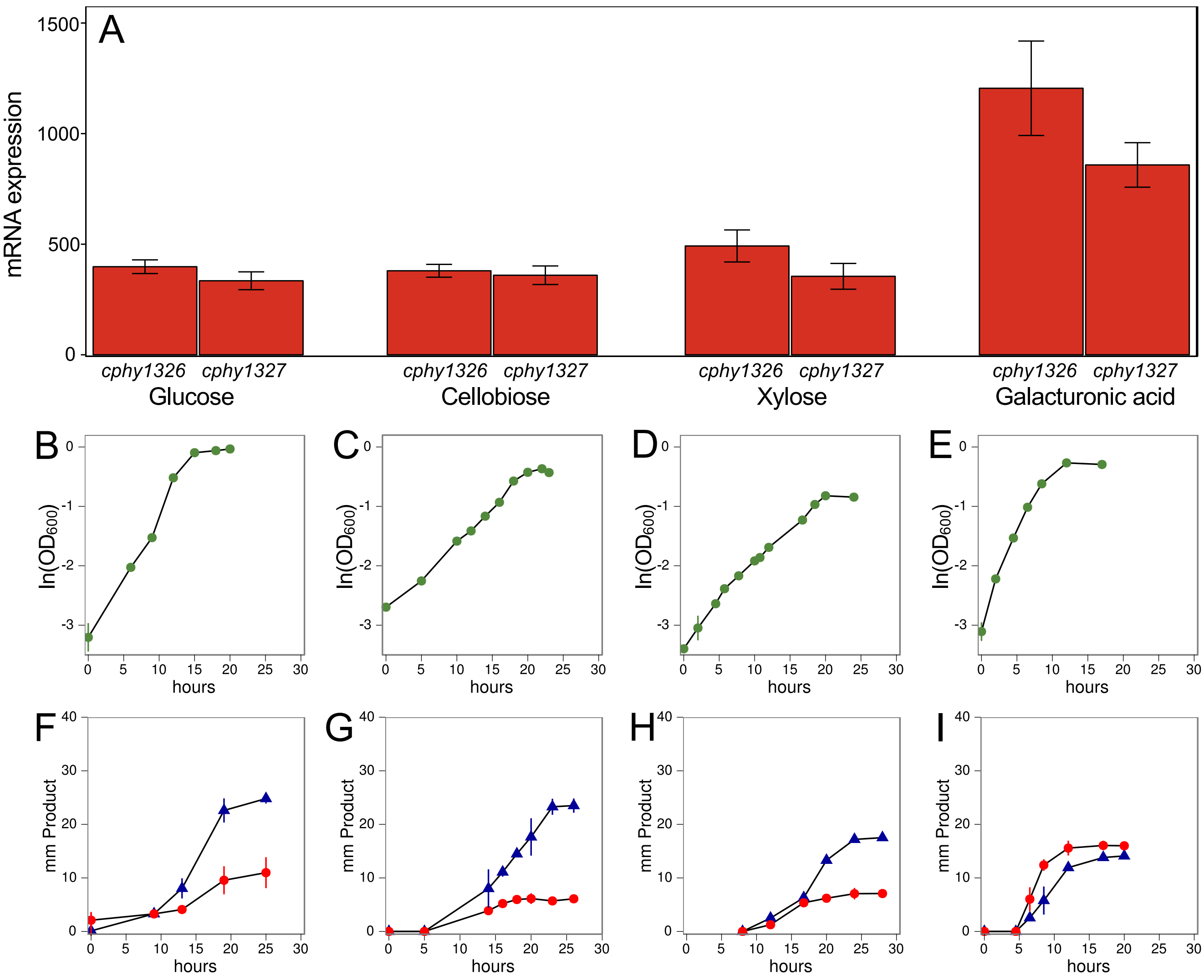

Supplementary Fig 9 *C. phytofermentans* increases expression of acetate synthesis genes and acetate formation on galacturonic acid. **A** mRNA expression of the *pta* (*cphy1326*) and *ackA* (*cphy1327*) acetate synthesis genes on glucose, cellobiose, xylose, and galacturonic acid. Expression is mean RPKM from duplicate cultures by RNA-seq; error bars are one standard deviation. Growth (OD_{600}) on **B** glucose, **C** cellobiose, **D** xylose, and **E** galacturonici acid. Accumulation of the two primary fermentation products, ethanol (blue triangles) and acetate (red circles), on **F** glucose, **G** cellobiose, **H** xylose, and **I** galacturonic acid. Data points for growth and fermentations are the mean of triplicate cultures; error bars are one standard deviation.

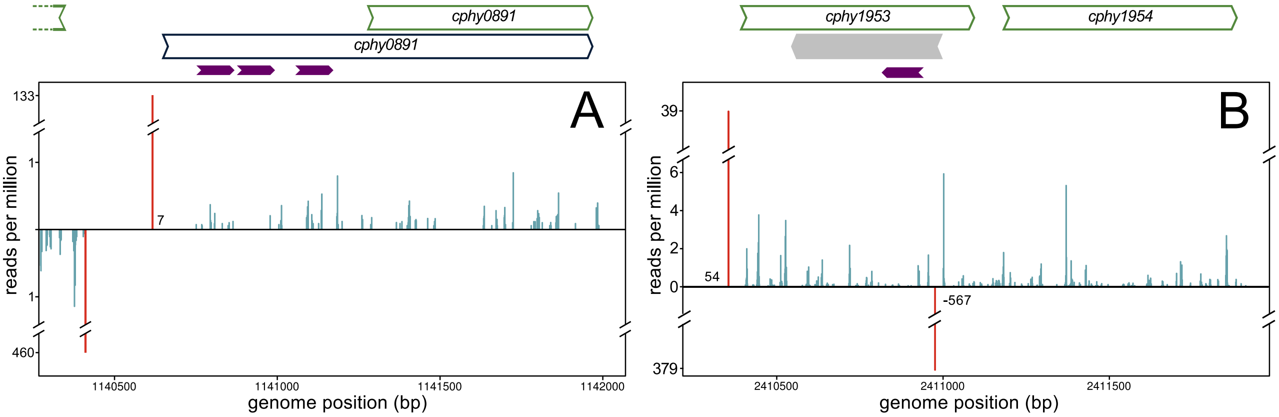

Supplementary Fig 10 Novel transcription units with TSS are supported by in-frame peptides to encode ORFs. **A** A primary TSS, RNA-seq reads, and 3 in-frame peptides on glucose support a 597 bp N-terminal extension to *cphy0891* that includes a pyrimidine 5'-nucleotidase domain. **B** The *cphy1953 comEA* gene is expressed based on a primary TSS and RNA-seq reads, but an antisense TSS and a peptide support an overlapping, antisense ORF. Expression on glucose is shown. Plots show number of reads starting at each genome position for Capp-Switch (red) and RNA-seq (blue). Numbers at bases of TSS peaks are distances to the **A** the start codon of *cphy0891* N-terminal extension, and **B** the *cphy1953* start codon. Above plots are the positions of genes in NCBI annotation (green), new genes (dark blue), and peptides detected by mass spectrometry (purple).

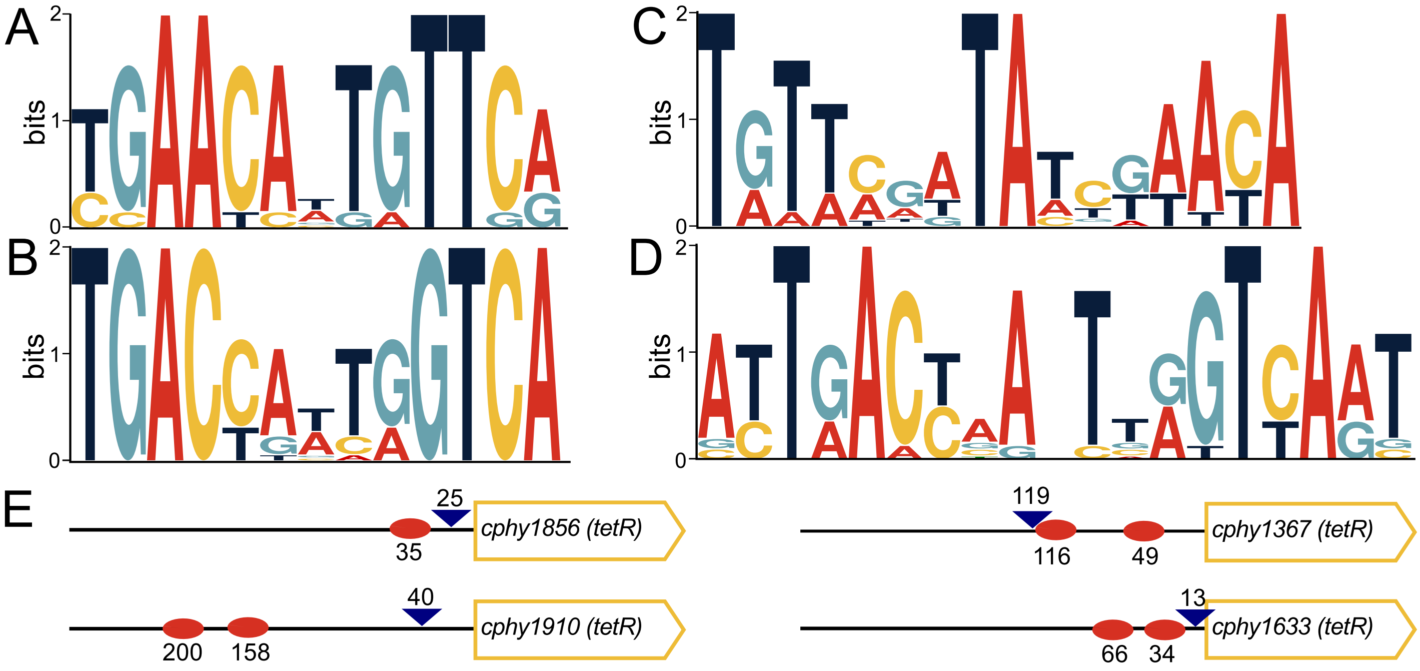

Supplementary Fig 11 *C. phytofermentans* TetR regulator genes **A** *cphy1856*, **B** *cphy1910*, **C** *cphy1367*, and **D** *cphy1633* share conserved, upstream palindromes with their orthologs representing putative TetO operator sites. **E** Relative positions of the palindrome motifs (red ovals) and primary TSS (blue triangles) upstream of tetR genes.

GENE	PROTEIN ANNOTATION	MOTIF POSITIONS	PRIMARY TSS POSITION
**Pectin Degradation**			
cphy2919	PL9: Family 9 pectate lyase. Degrades homogalacturonon (PMID 25393313)	181	145 (galacturonic acid)<br>98 (homogalacturonan
cphy3869	PL9: Family 9 pectate lyase. Degrades homogalacturonon (PMID 25393313)	165, 47	189 (galacturonic acid)<br>156 (homogalacturonan)
cphy1888	PL9: Family 9 pectate lyase. Degrades homogalacturonon (PMID 25393313)	245, 218, 146	184
cphy0203	GH105: Rhamnogalacturonyl hydrolase	127	-
**Galacturonic Acid Metabolism**			
cphy2534	KduD: 2-dehydro-3-deoxy-D-gluconate 5-dehydrogenase	237	199
cphy0054	KdgA: 2-keto-3-deoxy-6-phosphogluconate aldolase	128	125
cphy2741	UxaB: D-tagaturonate reductase	161	158
cphy0227	KdgK: 2-keto-3-deoxy-D-gluconate kinase		-
**Gene Regulation**			
cphy2742	LacI family transcription regulator	190, 166, 140	215
cphy0946	ECF subfamily RNA polymerase sigma-24 factor	39	-
cphy2734	AraC family transcriptional regulator	128	-
**Carbon Metabolism**			
cphy1326	phosphate acetyltransferase	201	139
cphy0651	pyruvate phosphate dikinase	227	90
cphy0367	alpha/beta hydrolase fold, lysophospholipase	217	67
**Other**			
cphy0368	cell wall hydrolase/autolysin, peptidoglycan aminohydrolase	75	-
cphy0381	MATE (Multi-antimicrobial extrusion) efflux family protein,<br>Na+-coupled multidrug efflux transporter	173, 77	-
cphy0431	extracellular solute-binding protein, amino acid transport	68	212
cphy0750	peptidylprolyl isomerase FKBP-type, catalyzes the cis-trans isomerisation of<br>Proline imidic peptide bonds in oligopeptides, protein folding	82	31
cphy0795	hypothetical	76	-
cphy1273	PspC: phage shock protein C	203, 122	-
cphy1325	hypothetical	159, -37	-
cphy3058	hypothetical	47	44

Supplementary Table 1 The 22 *C. phytofermentans* genes with putative Cphy2742 operator sites identified by searching -250 to +50 bp with respect to start codons for all genes in the *C. phytofermentans* genome using MEME with default parameters. Table includes gene name, annotation, distance of operator upstream of start codon, and distance of primary TSS upstream of start codon.

Sample	Total Reads	Mapped Reads	Percentage Mapped
Glucose 1	3,647,084	3,426,512	93.95
Glucose 2	2,942,008	2,836,086	96.40
Xylose 1	1,814,676	1,747,356	96.29
Xylose 2	2,194,098	2,094,373	95.45
Galacturonic acid 1	1,763,682	1,655,766	93.88
Galacturonic acid 2	1,137,648	1,077,362	94.70
Cellulose 1	1,556,286	1,461,136	93.89
Cellulose 2	1,895,814	1,729,337	91.22
Xylan 1	1,070,404	1,045,033	97.63
Xylan 2	2,286,760	2,238,769	97.90
Pectin (homogalacturonan) 1	3,424,780	3,278,318	95.72
Pectin (homogalacturonan) 2	1,612,046	1,477,097	91.63
Corn stover 1	462,936	404,111	87.29
Corn stover 2	500,090	453,625	90.71

Supplementary Table 2 Number of sequenced and mapped reads for paired-end Capp-Switch samples. Mapped reads are defined as those reported by Bowtie 2 to align with a single location in the *C. phytofermentans* genome (NC_010001.gbk). The 3 bp MMLV reverse transcriptase 3' non-template extension was removed from the 5’ end of reads in the first read set (R1) prior to mapping.

Primer	Sequence	Function
cphy1510_F	5' aagaccaagctttgaagaaccagtgccagaa 3'	PCR primer 1
cphy1510_RT	5' ggcttcatttcattctctaatgtg 3'	Reverse transcription
cphy2243_F	5' atacgtaagcttctaccatttgcccttgcata 3'	PCR primer 1
cphy2243_RT	5' tccgcatcttctacaccaaa 3'	Reverse transcription
cphy2876_F	5' tccaacaagctttggagcggagataacaacct 3'	PCR primer 1
cphy2876_RT	5' tcacctgtgtaagcgtggat 3'	Reverse transcription
Cphy3558_F (IntraS)	5' actacgaagctttgacaacatgctctgcatca 3'	PCR primer 1
Cphy3558_RT (IntraS)	5' tgacaacatgctctgcatca 3'	Reverse transcription
Cphy3558_F (primary TSS)	5' gatgttaagcttggacgcggtgtaggttgtag 3'	PCR primer 1
Cphy3558_RT (primary TSS)	5' gaacattaccggagcaaagc 3'	Reverse transcription
PCR_R	5' gaccacgcgtatcgatgtcgacttttttttttttttt[agc] 3'	PCR primer 2 (all genes)

Supplementary Table 3 Primers used for 5' RACE reverse transcription and PCR. Table includes primer sequences with restriction sites underlined.

Cphy_1883 (LacI) orthologs		
**Organism**	**Gene**	**Accession**
Halobacteroides halobius DSM 5150	Halha_1434	WP_015327101
Bacillus megaterium WSH 002	BMWSH_3101	WP_014460302
Bacillus megaterium DSM319	BMD_2102	WP_013082971
Thermobacillus composti KWC4	Theco_3366	WP_015256142
Clostridium saccharolyticum WM1	Closa_1189	WP_013271891
**Cphy_2467 (LacI) orthologs**		
**Organism**	**Gene**	**Accession**
Ruminococcus albus 7	Rumal_0460	WP_043550597
Clostridium saccharolyticum WM1	Closa_3860	WP_013274428
Eubacterium rectale ATCC 33656	EUBREC_1073	WP_012741936
Roseburia hominis A2 183	RHOM_03325	WP_014078832
Butyrivibrio proteoclasticus B316 Chromosome 1	bpr_I2446	WP_013281832
**Cphy_2742 (LacI) orthologs**		
**Organism**	**Gene**	**Accession**
Clostridium saccharolyticum WM1	Closa_1652	WP_013272339
Butyrivibrio proteoclasticus B316	bpr_I1592	ADL34329
Roseburia hominis A2 183	RHOM_08220	WP_014079796
Clostridium stercorarium subsp. Stercorarium DSM 853	Cst_c21170	WP_015359767
Clostridium lentocellum DSM 5427	Clole_1215	WP_013656242
**Cphy_1367 (TetR) orthologs**		
**Organism**	**Gene**	**Accession**
Desulfotomaculum gibsoniae DSM 7213	Desgi_2931	WP_006520819
Desulfotomaculum acetoxidans DSM 771	Dtox_2544	WP_015758039
Ruminococcus albus 7	Rumal_0807	WP_013497521
Selenomonas sputigena ATCC 35185	Selsp_1836	WP_006191032
Veillonella parvula DSM 2008	Vpar_0251	WP_008603003
**Cphy_1633 (TetR) orthologs**		
**Organism**	**Gene**	**Accession**
Clostridium cellulovorans 743B	Clocel_0472	WP_010074982
Brachyspira hyodysenteriae WA1	BHWA1_00546	WP_012670093
Brachyspira pilosicoli P43/6/78	BPP43_01525	WP_015273946
Eubacterium limosum KIST612	ELI_3977	WP_013382228
Coriobacterium glomerans PW2	Corgl_1663	WP_013709504
**Cphy_1856 (TetR) orthologs**		
**Organism**	**Gene**	**Accession**
Brachyspira murdochii DSM	Bmur_2301	WP_041750013
Brachyspira hyodysenteriae WA1	BHWA1_02346	WP_012671830
Eubacterium rectale ATCC 33656	EUBREC_2019	WP_012742856
Brachyspira pilosicoli P43/6/7	BPP43_09495	WP_015274758
Anaerococcus prevotii DSM 20548	Apre_1074	WP_015778004
**Cphy_1910 (TetR) orthologs**		
**Organism**	**Gene**	**Accession**
Butyrivibrio proteoclasticus B316	bpr_I2111	WP_013281498
Clostridium lentocellum DSM 5427	Clole_0058	WP_013655119
Eubacterium eligens ATCC 27750	EUBELI_00030	WP_041687853
Ruminococcus albus 7	Rumal_1063	WP_013497767
Clostridium botulinum B str. Eklund 17B	CLL_A0765	WP_012423768

Supplementary Table 4 *C. phytofermentans* LacI/GalR and TetR orthologs from related genomes used to identify putative operator motifs. Table includes organisms, NCBI accession numbers, and NCBI gene names.
